# Primary emotional systems and personality functioning in women with endometriosis receiving brief psychotherapy: a longitudinal study using ANPS and SWAP-200

**DOI:** 10.3389/fpsyg.2026.1782784

**Published:** 2026-04-08

**Authors:** Yvonne Radin, Elisa Farenga, Costanza Tersar, Giuseppe Ricci, Giovanni Di Lorenzo, Andrea Clarici

**Affiliations:** 1 S.C.U. Obstetrics and Gynaecology Clinic, Institute for Maternal and Child Health, IRCCS “Burlo Garofolo”, Trieste, Italy; 2 Department of Medical, Surgical and Health Sciences, Cattinara Hospital, University of Trieste (UniTS), Trieste, Italy

**Keywords:** affective neuroscience personality scales, ANPs, chronic pelvic pain, dysmenorrhea, dyspareunia, endometriosis

## Abstract

**Background:**

Endometriosis is a chronic gynecological condition often associated with severe pelvic pain, reduced quality of life, and significant psychological and sexual distress. Although psychological factors are known to influence pain and adaptation, psychodynamic and personality-informed interventions remain rarely explored. It is also unclear whether endometriosis shares an emotional signature with other conditions involving psychic pain, such as functional neurological disorder (FND).

**Methods:**

Women attending a tertiary endometriosis clinic in Trieste, Italy, who reported psychological or sexual distress were offered a psycho-sexual orientation interview and, if they agreed, a brief psychotherapeutic pathway. Seventeen women with endometriosis (7 operated, 10 non-operated) completed a 10-session intervention and an assessment protocol. Primary emotional dispositions were assessed with the Affective Neuroscience Personality Scales (ANPS) at pre-treatment, post-treatment, and 2-month follow-up. Personality configuration was evaluated at baseline with the Shedler Westen Assessment Procedure (SWAP-200). Repeated-measures analyses tested changes in ANPS scales, and correlations examined links between ANPS and SWAP-200 indices.

**Results:**

Participants showed high medical and psychiatric comorbidity and a predominantly high-functioning depressive–obsessive personality profile. Across time, only the ANPS SADNESS scale showed a significant reduction from baseline to follow-up, while other primary emotions remained stable. More high-functioning obsessive features were associated with adaptive emotional profiles, whereas more dysregulated features correlated with higher negative affect.

**Conclusion:**

A brief psychodynamic intervention integrated into gynecological care was associated with a selective reduction in chronic dysphoric affect. These exploratory convergent findings across endometriosis and FND might suggest a shared mechanism of affect regulation relevant to complex pain-related conditions.

## 1 Introduction

Endometriosis is a chronic gynecological condition affecting an estimated 5–10% of women of reproductive age and a leading cause of chronic pelvic pain (CPP), dysmenorrhea, dyspareunia, and infertility. These symptoms markedly impair daily functioning, sexuality, and health-related quality of life (HRQoL). Symptomatic endometriosis, particularly when CPP is present, is consistently associated with lower HRQoL and poorer mental health compared with asymptomatic endometriosis and healthy controls (Facchin et al., 2015; Farenga et al., 2024). Pain characteristics are clinically relevant: non-menstrual pelvic pain, neuropathic-like components, and pain catastrophizing are strongly linked to disability and diminished HRQoL (Dolci et al., 2025).

Beyond physical suffering, endometriosis entails a substantial psychological burden. Meta-analytic evidence indicates higher depressive and anxiety symptoms in women with endometriosis than in controls (Yang et al., 2025; Szypłowska et al., 2023), with greater distress in the presence of more severe or multisite pain and associated fatigue, sleep problems, and reduced mental HRQoL (Szypłowska et al., 2023). Qualitative studies further describe persistent challenges in integrating pain, infertility concerns, and experiences of loss or injustice into identity, relationships, and future plans (Farenga et al., 2024).

These observations align with chronic pain research conceptualizing pain and emotional distress as tightly coupled processes. Emotional difficulties (e.g., depression, anxiety, alexithymia, maladaptive regulation) both predict and are exacerbated by chronic pain, contributing to hypervigilance, catastrophizing, and functional impairment (Ravyts et al., 2024). In endometriosis, emotion regulation strategies, including acceptance, mindfulness, and self-compassion, as well as rumination and catastrophizing, are systematically associated with pain intensity, depressive symptoms, and quality of life (Carvalho et al., 2025). Accordingly, endometriosis-related pain can be understood as not only somatic but also affective and relational, particularly in the domains of sexuality, body image, and fertility, where shame, anger, and grief may intersect with couple intimacy and identity (Farenga et al., 2024; Dolci et al., 2025). Intervention studies suggest that psychological treatments (e.g., mindfulness-based and other structured programs) can reduce pain, improve pain-related cognitions, and enhance mental-health-related quality of life in endometriosis (Moreira et al., 2023; Carvalho et al., 2025), yet psychodynamic or personality-informed approaches remain comparatively underexplored.

A parallel literature has developed in functional neurological disorder (FND), characterized by neurological symptoms incongruent with known disease and increasingly framed within predictive coding/free-energy models linking symptom expression to aberrant expectations, salience attribution, and emotional-relational conflict (Espay et al., 2018). FND is frequently associated with chronic pain, anxiety and depression, trauma histories, and emotion regulation difficulties, and psychotherapy is considered a core component of multidisciplinary care (Espay et al., 2018; Myers et al., 2021; Russell et al., 2022).

In a previous longitudinal study conducted at the same institution, we delivered a 10-session psychotherapeutic intervention to FND patients and used the Affective Neuroscience Personality Scales (ANPS; Panksepp and Davis, 2012) and the Shedler–Westen Assessment Procedure (SWAP-200; Shedler and Westen, 2004) to assess primary emotional systems and personality functioning (Radin et al., 2025). We found a selective reduction in ANPS SADNESS over time, relative stability in other primary emotions and personality configurations, and clinically meaningful associations between internalizing personality profiles and heightened negative affect. For clarity, we use SADNESS when referring to the ANPS self-report scale and PANIC/GRIEF when referring to the underlying affective neuroscience system of separation distress.

Although endometriosis and FND differ in pathophysiology, they share clinically relevant features: persistent, often disabling bodily symptoms; psychiatric comorbidity; and difficulties in emotion regulation and mentalizing bodily states (Szypłowska et al., 2023; Espay et al., 2018; Radin et al., 2025). Building on this conceptual convergence, we integrated a psycho-sexual orientation interview and a 10-session individual psychotherapeutic intervention into routine care within a tertiary endometriosis clinic (IRCCS Burlo Garofolo, Trieste, Italy) for women reporting psychological or sexual distress related to endometriosis. Using the same ANPS and SWAP-200 instruments as in our FND study, we aimed to: (a) describe sociodemographic, clinical, affective, and personality characteristics of women referred to psychological care; (b) examine longitudinal changes in primary emotional systems across pre-treatment, post-treatment, and 2-month follow-up; and (c) explore correspondence between self-reported affective profiles (ANPS) and clinician-rated personality configurations (SWAP-200). In a secondary, exploratory step, we relate these findings descriptively to our previously published FND cohort to generate hypotheses about potential cross-diagnostic affective targets.

## 2 Materials and methods

### 2.1 Setting and participants

Participants were recruited from October 2022 to March 2024 at the IRCCS Burlo Garofolo in Trieste, Italy, within the gynaecology department’s endometriosis clinic, which runs two dedicated outpatient sessions per month. During these specialized clinics, the gynaecologist systematically explored pain, reproductive issues and the presence of psychological and sexual distress. Women with a diagnosis of endometriosis who reported significant emotional or relational difficulties related to their condition were informed about the possibility of taking part in a psychological project.

A total of 28 women with endometriosis (12 previously operated, 16 non-operated) were initially approached after their endometriosis clinic visit. They were offered a psycho-sexual orientation interview with a psychologist with specific training in sexology and, where appropriate, a subsequent 10-session individual psychotherapeutic intervention. Of these, 17 women (7 operated, 10 non-operated) accepted the orientation interview, agreed to start psychotherapy, completed all 10 sessions and provided full data on the ANPS and SWAP-200. These 17 women constitute the analytic sample for the present study.

Among the 11 women who did not proceed to the 10-session intervention and/or complete the assessment, reasons reported during routine clinical contact were heterogeneous and primarily practical (e.g., low readiness for a structured commitment, language barriers, work or family constraints), as well as ambivalence or reluctance toward psychotherapy.

### 2.2 Procedure

After the gynaecological consultation, eligible women were invited to a psycho-sexual orientation interview. This orientation interview was a qualitative intake session focused on (i) sexual functioning and dyspareunia-related concerns, (ii) relational and intimacy issues, and (iii) emotional responses to endometriosis. Based on this meeting, women could choose whether to proceed with the psychological pathway.

Those who agreed received a 10-session individual psychotherapeutic intervention delivered by a psychotherapist trained in psychodynamic and relational approaches. The intervention focused on helping patients recognize and name emotions related to pain, infertility concerns, relational difficulties and the impact of endometriosis on identity and daily life, with attention to attachment dynamics and bodily experience. At intake, participants were asked about prior psychological treatments; 8/17 reported previous psychological support, typically brief and heterogeneous (e.g., adolescent counselling, online support, CBT-oriented consultations), and not specifically related to endometriosis. Furthermore, no participant reported concurrent psychotherapy outside the hospital service during this intervention.

The intervention was delivered within the hospital service by three licensed psychotherapist trained in psychodynamic approaches. The psychological support was grounded in two complementary theoretical frameworks. First, we adopted a predictive-processing perspective in which symptoms are understood as meaningful expressions of affective activation and adaptive action tendencies, linking emotional arousal to the individual’s coping responses (Solms and Friston, 2018; Solms, 2021). Second, the intervention was informed by Kernberg’s Transference-Focused Psychotherapy (TFP) model, which emphasizes identifying and working through recurrent internalized relationship patterns as they emerge in the therapeutic relationship (i.e., transference), in order to clarify affective conflicts and promote more integrated emotion regulation (Kernberg, 1984; Kernberg and Caligor, 2005).

Therapist allocation was based on a pre-specified allocation list designed to maintain balance across the three clinicians within routine clinical practice. The sequence was generated using repeated balanced sets of therapist assignments arranged in randomized order, and patients who accepted the psychotherapy pathway were assigned sequentially to the next available therapist indicated on the list. Because this procedure relied on repeated balanced assignment sets rather than on a fixed final sample, it did not require the total number of patients to be specified in advance. This approach was adopted to combine a non-discretionary allocation process with a balanced distribution of cases across therapists. Twice a month, treatment delivery was supported by regular clinical supervision and routine case discussion within the service to enhance consistency.

The Affective Neuroscience Personality Scales (ANPS) were administered at three time points: pre-treatment (T0), post-treatment (T1; after the 10th session), and at 2-month follow-up (T2). The SWAP-200 was completed by the treating psychotherapist (or a clinician with full access to the clinical material) at baseline (T0).

Ethical approval was obtained from the local Ethics Committee of IRCCS Burlo Garofolo, and all participants provided written informed consent.

### 2.3 Measures

#### 2.3.1 Clinical and reproductive variables

Clinical variables were extracted from routine hospital records and intake documentation collected during the gynaecological first visits; psychiatric comorbidity reflects what was documented in the medical chart based on clinical history taken by non-psychiatric providers.

Specifically, we extracted age, surgical status (operated vs. non-operated for endometriosis), medical comorbidities, psychiatric comorbidities, and detailed pain symptoms (dysmenorrhea, chronic pelvic pain, ovulatory pain, dyspareunia, dyschezia). Reproductive history included previous pregnancies, post-surgical pregnancies (for operated women), current attempts to conceive and history of visits to medically assisted reproduction (PMA) services. “Operated” refers to participants with a history of surgical treatment for endometriosis (e.g., laparoscopic excision/ablation) prior to entering the psychological pathway, whereas “non-operated” refers to participants with no prior endometriosis surgery.

#### 2.3.2 Affective Neuroscience Personality Scales (ANPS)

The ANPS is a self-report questionnaire derived from Panksepp’s affective neuroscience framework, assessing six primary emotional systems—SEEKING, PLAY, CARE, FEAR, ANGER, SADNESS—and one secondary system, LUST (Panksepp and Davis, 2012). Higher scores indicate greater disposition to experience and express the corresponding affective system. The ANPS has shown good reliability and validity across clinical and non-clinical samples and has been used in both endometriosis and FND research (Panksepp and Davis, 2012; Radin et al., 2025).

#### 2.3.3 Shedler–Westen Assessment Procedure (SWAP-200)

The SWAP-200 is a clinician-rated, Q-sort instrument designed to assess personality syndromes, personality disorder prototypes and a global index of psychological health/structural functioning (Shedler and Westen, 2004). It yields T-scores for DSM/ICD personality disorder prototypes (PDT scales) and empirically derived Q-factor scales (QT scales), including high-functioning, depressive–high-functioning, obsessive, dysregulated–borderline, dependent, avoidant and others. T-scores ≥ 60 are typically considered clinically salient elevations. The SWAP-200 has been widely validated and used in both personality and psychotherapy outcome research (Lingiardi and McWilliams, 2017; Westen and Shedler, 1999).

### 2.4 Statistical analysis

Descriptive statistics were calculated for sociodemographic, clinical and reproductive variables in the analytic sample (*N* = 17), and by surgical status (Table 1).

**Table 1:** Sociodemographic, clinical, reproductive, and psychological characteristics of the analytic sample.

Characteristic	Total sample (*N* = 17)	Operated for endometriosis (*n* = 7)	Non-operated (*n* = 10)
Age, years, *M* (SD)^a^	30.7 (7.6)	32.8 (9.7)	29.3 (5.9)
Previous surgery for endometriosis, *n* (%)	7 (41.2%)	7 (100%)	0 (0%)
Any medical comorbidity, *n* (%)	14 (82.4%)	5 (71.4%)	9 (90.0%)
Psychiatric comorbidity, *n* (%)	5 (29.4%)	0 (0.0%)	5 (50.0%)
Pelvic pain symptoms, *n* (%)			
Dysmenorrhea	5 (29.4%)	0 (0.0%)	5 (50.0%)
Chronic pelvic pain	6 (35.3%)	0 (0.0%)	6 (60.0%)
Ovulatory pain	7 (41.2%)	5 (71.4%)	2 (20.0%)
Dyspareunia	12 (70.6%)	5 (71.4%)	7 (70.0%)
Dyschezia	6 (35.3%)	3 (42.9%)	3 (30.0%)

Values are expressed as mean and standard deviation (*M*, SD) or frequency and percentage, as appropriate.

a For non-operated patients, age refers to age at first gynecological visit; for operated patients, age refers to age at the time of endometriosis surgery.

To examine change in primary emotional systems over time, we conducted one-way repeated-measures ANOVAs on each ANPS scale (SEEKING, PLAY, CARE, FEAR, ANGER, SADNESS, LUST), with Time (T0, T1, T2) as a within-subject factor. We report *F* values, associated *p* values and partial eta squared (η_p_^2^) as a measure of effect size. Where a significant main effect of Time emerged, we conducted *post-hoc* paired *t*-tests (T0–T1, T0–T2, T1–T2) with within-subject Cohen’s *d* to quantify the magnitude of change.

SWAP-200 PDT and QT T-scores were summarized at baseline, and we explored Pearson correlations among SWAP indices and between SWAP indices and baseline ANPS scales (T0). We used Pearson’s correlation coefficient because both ANPS and SWAP scores are continuous psychometric measures, approximately normally distributed in this sample, and our aim was to quantify the strength and direction of linear associations between them. We highlight moderate to large associations (|*r*| ≳ 0.50), recognizing that the small sample size limits statistical power and precision. All analyses were conducted in Python (Python Software Foundation, 2020) using NumPy (Harris et al., 2020), pandas (McKinney, 2010), and matplotlib (Hunter, 2007).

Finally, we interpret the endometriosis findings in relation to our previously published FND cohort, which used the same assessment battery and 10-session psychotherapeutic format (Radin et al., 2025). No formal Time × Group analysis was possible because raw FND data were not pooled with the current dataset; comparisons are therefore exploratory and descriptive.

## 3 Results

### 3.1 Sociodemographic and clinical characteristics

Table 1 summarizes the sociodemographic, clinical and reproductive characteristics of the analytic sample. The 17 women had a mean age of 30.7 years (SD = 7.6); operated women were slightly older (*M* = 32.8, SD = 9.7; age at surgery) than non-operated women (M = 29.3, SD = 5.9; age at first gynaecological visit). Almost all patients (16/17, 94.1%) presented at least one relevant medical comorbidity, and 5/17 (29.4%) had a documented psychiatric comorbidity, more frequently among non-operated women (5/10, 50.0%) than in the operated subgroup (0/7, 0.0%).

Pelvic pain symptoms were highly prevalent: dyspareunia was reported by 12 women (70.6%), chronic pelvic pain by 6 (35.3%), dysmenorrhea by 5 (29.4%), ovulatory pain by 7 (41.2%) and dyschezia by 6 (35.3%). Four women (23.5%) had experienced at least one previous pregnancy, with a higher proportion in the operated group (3/7, 42.9%) than in the non-operated group (1/10, 10.0%); one operated woman (14.3%) had a spontaneous pregnancy after surgery. One non-operated patient (5.9% of the total sample) reported currently seeking pregnancy and had consulted a PMA service.

By design, all 17 women accepted the psycho-sexual orientation interview, agreed to the 10-session psychotherapeutic pathway and completed all sessions, with no dropouts in this analytic sample.

### 3.2 ANPS primary emotion systems over time

Baseline (T0) ANPS scores showed elevated negative affect compared to normative data reported in the literature, with mean scores of 28.5 for SEEKING (SD = 7.3), 22.9 for PLAY (5.0), 29.9 for CARE (5.1), 28.2 for FEAR (7.4), 23.1 for ANGER (7.0), 27.0 for SADNESS (5.3) and 24.9 for LUST (7.4).

**Figure 1:**
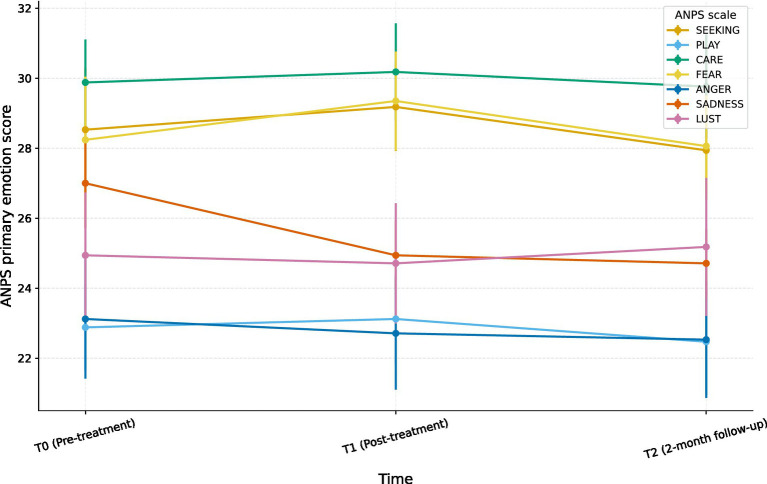
Mean ANPS primary emotion scores across time. Lines represent mean scores on the Affective Neuroscience Personality Scales (ANPS) primary emotion dimensions (SEEKING, PLAY, CARE, FEAR, ANGER, SADNESS, LUST) at pre-treatment (T0), post-treatment (T1), and 2-month follow-up (T2); error bars indicate ±1 standard error of the mean (SEM). A significant main effect of time emerged only for SADNESS, *F*(2, 32) = 3.45, *p* = 0.044, partial η^2^ = 0.18; no significant time effects were found for the other scales.

The repeated-measures ANOVAs (Table 2; Figure 1) indicated a significant main effect of Time only for SADNESS:

**Table 2:** Repeated-measures ANOVA of ANPS primary emotion scores at pre-treatment, post-treatment, and 2-month follow-up.

ANPS scale	T0 *M* (SD)	T1 *M* (SD)	T2 *M* (SD)	*F*(2, 32)	*p*	η_p_^2^
SEEKING	28.53 (7.30)	29.18 (5.19)	27.94 (5.81)	0.77	0.472	0.05
PLAY	22.88 (5.02)	23.12 (5.38)	22.47 (6.27)	0.23	0.800	0.01
CARE	29.88 (5.06)	30.18 (5.73)	29.76 (6.12)	0.15	0.863	0.01
FEAR	28.24 (7.44)	29.35 (5.82)	28.06 (5.98)	0.81	0.456	0.05
ANGER	23.12 (7.03)	22.71 (6.63)	22.53 (6.87)	0.18	0.834	0.01
SADNESS	27.00 (5.29)	24.94 (4.10)	24.71 (4.04)	3.45	0.044	0.18
LUST	24.94 (7.40)	24.71 (7.09)	25.18 (8.13)	0.06	0.941	0.00

Values are means with standard deviations in parentheses. Results are from one-way repeated-measures ANOVAs with Time (T0 = pre-treatment, T1 = post-treatment, T2 = 2-month follow-up) as a within-subject factor. η_p_^2^ = partial eta squared.

SADNESS: *F*(2, 32) = 3.45, *p* = 0.044, η_p_^2^ ≈ 0.18.

- T0: *M* = 27.00 (SD = 5.29)
- T1: *M* = 24.94 (SD = 4.10)
- T2: *M* = 24.71 (SD = 4.04)

All other ANPS scales did not show significant time effects (*ps* > 0.45), with small *F* values and η_p_^2^ in the small-to-negligible range.

*Post-hoc* paired comparisons for SADNESS showed:

- T0 vs. T1: *t*(16) = 1.87, *p* ≈ 0.080, *d* ≈ 0.45
- T0 vs. T2: *t*(16) = 2.40, *p* ≈ 0.029, *d* ≈ 0.58
- T1 vs. T2: *t*(16) = 0.29, *p* ≈ 0.77, *d* ≈ 0.07

Without correction for multiple comparisons, SADNESS decreased significantly from baseline to 2-month follow-up with a medium within-subject effect size, whereas the T0–T1 contrast was in the expected direction but reached only trend-level significance. Scores were essentially stable between T1 and T2, suggesting that the sssswreduction achieved by the end of treatment was maintained at follow-up. Applying a strict Bonferroni correction renders the T0–T2 comparison borderline, so the effect should be interpreted as suggestive but clinically meaningful given the small sample size.

Taken together, these findings indicate a selective and modest decrease in SADNESS over time during the intervention period, whereas the other ANPS scales remained stable.

### 3.3 SWAP-200 personality profile

SWAP-200 clinician ratings indicated a personality configuration characterized by high psychological functioning and internalizing depressive–obsessive traits, with limited evidence of classic personality disorder.

High-Functioning (PDT/QT) T-scores were elevated (*M* ≈ 62.9, SD ≈ 11.1), with 11/17 women (≈ 65%) scoring ≥ 60. Obsessive Q-factor scores were similarly high (*M* ≈ 60.6, SD ≈ 13.4; 11/17 ≥ 60), and the Depressive–High Functioning Q-factor was also elevated (*M* ≈ 61.0, SD ≈ 8.7; 10/17 ≥ 60). In contrast, personality disorder prototype scales for paranoid, schizoid/schizotypal, antisocial, borderline, histrionic, narcissistic, avoidant, dependent and obsessive PDs all had mean T-scores in the 40s, with few or no values above 60. Emotional dysregulation, narcissistic and antisocial Q-factors were, on average, in the low 40s, with very few patients in the clinically elevated range.

Overall, the SWAP-200 profile depicts a sample of women who are psychologically high functioning, conscientious and organized, with internalizing depressive and obsessive features but relatively little overt personality disorder in the classic borderline/antisocial sense (see Tables 3, 4).

**Table 3:** Correlations between baseline ANPS primary emotion scales and SWAP-200 Q-factor scores (QT) (*N* = 17).

ANPS	Antis. QT	Schiz. QT	Paran. QT	Obsess. QT	HistrionQT	Narciss. QT	Avoid. QT	Depress.–High-Funct. QT	Dysreg. Emotional QT	Depend. QT	HostilQT	High-Funct. QT
SEEKING	−0.44	−0.20	−0.12	0.67**	−0.19	0.44	0.14	0.41	−0.58*	−0.29	−0.11	0.52*
PLAY	−0.54*	−0.25	−0.50*	0.49*	0.23	0.18	0.12	0.34	−0.61**	0.23	−0.32	0.40
CARE	−0.67**	0.14	−0.45	0.35	0.15	0.00	0.31	0.16	−0.44	0.10	−0.39	0.26
FEAR	0.53*	0.27	0.38	−0.32	−0.03	0.19	0.40	−0.25	0.55*	0.44	0.59*	−0.47
ANGER	0.75***	0.08	0.40	−0.66**	−0.04	0.37	0.00	−0.45	0.54*	0.49*	0.58*	−0.60*
SADNESS	0.25	0.13	−0.01	−0.38	0.08	0.14	0.23	−0.26	0.33	0.59*	0.13	−0.41
LUST	−0.50*	−0.10	−0.40	0.49*	0.04	−0.06	0.06	0.18	−0.46	−0.09	−0.48	0.30

Values are Pearson’s r. ANPS scores refer to baseline (T0) primary emotion scales. QT = SWAP-200 empirically derived Q-factor scales. *p* < 0.05*, *p* < 0.01**, *p* < 0.001*** (two-tailed).

**Table 4:** Correlations between baseline ANPS primary emotion scales and SWAP-200 PD prototype scores (PDT) (*N* = 17).

ANPS	Paranoid PDT	Schizoid PDT	Schizotypal PDT	Antisocial PDT	Borderline PDT	Histrionic PDT	Narcissistic PDT	Avoidant PDT	Dependent PDT	Obsessive PDT	High-Functioning PDT
SEEKING	−0.11	−0.12	−0.39	−0.36	−0.60*	−0.52*	0.12	0.02	−0.04	0.77***	0.52*
PLAY	−0.42	−0.23	−0.37	−0.54*	−0.48*	−0.16	−0.10	−0.07	0.36	0.35	0.40
CARE	−0.44	0.06	−0.01	−0.64**	−0.46	−0.18	−0.27	0.06	0.35	0.27	0.26
FEAR	0.49*	0.42	0.36	0.30	0.67**	0.41	0.18	0.63**	0.51*	−0.05	−0.47
ANGER	0.53*	0.21	0.24	0.67**	0.70**	0.56*	0.43	0.30	0.19	−0.37	−0.60*
SADNESS	0.14	0.18	0.27	0.18	0.47	0.35	0.00	0.31	0.43	−0.30	−0.41
LUST	−0.34	−0.13	−0.27	−0.44	−0.53*	−0.29	−0.22	−0.16	0.02	0.40	0.30

Values are Pearson’s r. ANPS scores refer to baseline (T0) primary emotion scales. PDT = SWAP-200 personality disorder prototype *T*-scores. *p* < 0.05*, *p* < 0.01**, *p* < 0.001***.

### 3.4 Associations between ANPS and SWAP-200

Correlations within SWAP-200 indices largely reproduced patterns described in validation studies. High-Functioning scales were strongly negatively correlated with most personality disorder prototypes and dysregulated Q-factors (e.g., *r* ≈ −0.81 with Borderline PDT; *r* ≈ −0.81 with Dysregulated–Emotional QT) and strongly positively correlated with Obsessive and Depressive–High Functioning Q-factors (e.g., *r* ≈ +0.91 and +0.95 respectively), consistent with their interpretation as a general index of psychological health compatible with certain high-functioning depressive or obsessive traits.

Examining cross-method associations, higher SWAP High-Functioning/Obsessive scores were associated with greater ANPS SEEKING and PLAY and lower FEAR, ANGER and SADNESS, indicating a more engaged, exploratory and erotically responsive affective style with less chronic negative affect. Conversely, borderline and dysregulated SWAP profiles (though relatively uncommon in this sample) were associated with higher ANPS FEAR, ANGER and SADNESS and lower SEEKING, PLAY and LUST, a pattern consistent with a dysphoric, negative-affectivity cluster. Dependent and avoidant features correlated positively with ANPS FEAR and SADNESS, reflecting a classic internalizing spectrum, whereas ANPS ANGER was most strongly related to antisocial and hostile Q-factors.

Given the small sample size, these correlations should be viewed as exploratory and descriptive. Nonetheless, they closely parallel co-occurrence patterns reported in SWAP-200 taxonomy studies, while highlighting a specific subgroup of high-functioning, obsessively organized women with depressive/internalizing affective styles in the context of chronic endometriosis.

## 4 Discussion

### 4.1 Main findings

This brief research report examined primary emotional systems and personality functioning in women with endometriosis who completed a 10-session psychotherapeutic intervention integrated into a tertiary endometriosis clinic. Three main findings emerged.

First, at baseline the sample was clinically complex: nearly all women had medical comorbidities, about one third had psychiatric comorbidities, and pelvic pain, especially dyspareunia and chronic pelvic pain, was highly prevalent. SWAP-200 ratings showed a predominantly high-functioning depressive–obsessive profile (elevated Q-factors in this personality configuration) with relatively low levels of classic personality disorder, suggesting structurally resilient and conscientious patients burdened by chronic grief, worry, and internalizing distress. This quantitative profile aligned with interview-based clinical impressions: many women presented as highly efficient and self-disciplined yet struggled with guilt about perceived sexual or reproductive “dysfunction.” This configuration may reflect a compensatory pattern in which emotional pain and vulnerability are managed through overcontrol and performance rather than reliance on others.

Second, longitudinal ANPS results indicated a selective reduction in SADNESS (PANIC/GRIEF) across treatment and follow-up (medium effect size; maintained at 2 months), while the other ANPS scales remained stable. This suggests that the intervention might have primarily modulated chronic dysphoric affect rather than broadly reshaping motivational systems; given persistent nociceptive input, medical uncertainty, and fertility concerns in endometriosis, a reduction in hopelessness, resignation, and grief may be more realistic than short-term changes in approach- or threat-related systems. Given the small sample, modest effect magnitude, multiple testing, and absence of a control group, changes in SADNESS cannot be causally attributed to psychotherapy. Alternative explanations include nonspecific therapeutic factors (e.g., alliance/support), natural adaptation over time, and concurrent medical care.

From a clinical perspective, psychologists often observed a combination of strong control needs and pervasive loneliness, shaped and reinforced by relational and healthcare-context factors. In the context of chronic pain, uncertainty, and repeated clinical encounters, some patients may adopt a controlled, self-reliant stance to maintain functioning, especially when experiencing limited validation or fragmented support; relational strain linked to dyspareunia and fertility concerns may further reinforce “managing alone” strategies. Patients frequently described difficulty relying on others, with sparse or emotionally “flat” relational narratives despite intense suffering. A need for closeness coexisted with fears of dependence (feeling “at the mercy” of the other), activating self-sufficiency and control. This qualitative pattern converged with the quantitative findings (high-functioning obsessive–depressive SWAP-200 profile, low overt personality disorder, and selective ANPS SADNESS reduction), suggesting that brief psychotherapy may help patients name and share grief and isolation and modestly reduce reliance on rigid self-control.

Third, in this sample, baseline (T0) cross-method associations between ANPS and SWAP-200 were coherent and clinically meaningful. High-functioning/obsessive profiles were linked to higher SEEKING/PLAY and lower negative affect, whereas borderline/dysregulated profiles were associated with higher FEAR/ANGER/SADNESS and lower SEEKING/PLAY/LUST; dependent and avoidant features tracked with FEAR and SADNESS. Although exploratory, these associations provide convergent information for case formulation, suggesting potentially different salient affective targets (e.g., PANIC/GRIEF-related dysphoria vs. threat-related affect) within integrated endometriosis care.

### 4.2 Comparison with functional neurological disorder

The comparison with our prior FND cohort is presented as an exploratory, descriptive secondary interpretation, not as a pre-specified cross-diagnostic analysis. In that cohort, the same 10-session psychotherapeutic intervention led to a significant reduction in ANPS SADNESS [*F*(2, 52) = 5.20, *p* = 0.009, partial η^2^ ≈ 0.17], with no significant time effects on other primary emotion systems (Radin et al., 2025). In the present endometriosis sample, SADNESS showed a similar medium-sized reduction [*F*(2, 32) = 3.45, *p* = 0.044, partial η^2^ ≈ 0.18], again as the only ANPS dimension with a significant time effect.

Conceptually, PANIC/GRIEF-related dysphoria may be clinically salient across distinct pain-related conditions because chronic symptoms can amplify loss-, separation-, and hopelessness-related affect, which in turn may increase bodily vigilance and symptom-related distress. Brief psychodynamic support may preferentially influence this domain by providing a structured relational context in which grief−/loss-related affect can be recognized, mentalized, and shared.

At the same time, baseline affective profiles seem to differ. Patients with endometriosis showed moderately higher SEEKING, FEAR and ANGER, and somewhat higher SADNESS, compared with the FND sample, whereas PLAY, CARE and LUST were broadly similar. FND patients appeared more inhibited, with lower SEEKING and ANGER despite elevated FEAR, suggesting a more withdrawal-prone style. By contrast, women with endometriosis appeared more “activated,” combining higher drive and exploratory motivation with strong fear and anger about a chronic organic illness. Personality profiles also differed: FND patients were characterized by more dependent/avoidant and dysregulated configurations, while the endometriosis group showed a more consistently high-functioning depressive–obsessive pattern.

Finally, it shall be noted that any cross-diagnostic comparisons with the FND cohort are descriptive and hypothesis-generating only, given the absence of pooled data and statistical control. In the endometriosis cohort, PANIC/GRIEF-related distress occurs alongside persistent nociceptive input and relatively intact functioning, indicating that brief psychodynamic support may be particularly relevant for helping patients articulate grief and loss, integrate illness into their life narrative, and address impacts on identity, sexuality, and relationships.

### 4.3 Clinical implications

Clinically, these findings reinforce the value of embedding psychological and sexological expertise directly within specialized endometriosis care. The recruitment model used here where the gynaecologist identifies psychological and sexual distress and offers a psycho-sexual orientation interview, allowing women already in contact with the gynaecological service to access a structured psychotherapeutic space. The fact that all 17 women in the analytic sample completed the 10-session pathway suggests that such an integrated approach is feasible and acceptable in a tertiary endometriosis clinic.

The selective reduction in SADNESS underscores the importance of targeting grief, loss and chronic discouragement in psychotherapeutic work with women with endometriosis, alongside more traditional pain management strategies. Interventions that help patients articulate and share the emotional meaning of pain, infertility concerns and repeated medical procedures, within a stable therapeutic relationship, may complement biomedical treatments and contribute to more comprehensive care.

In the exploratory comparison with our previously published FND cohort, the observed associations between personality functioning and affective patterns suggest that tailoring the focus of brief psychological support to broad personality configurations may be a useful hypothesis for future testing. For example, high-functioning depressive–obsessive presentations may warrant greater attention to self-criticism and overcontrol, whereas more dysregulated or dependent patterns may call for stronger emphasis on emotion regulation, attachment security and boundary setting.

In this endometriosis context, where emotional distress unfolds alongside persistent nociceptive input and often relatively intact functioning, the clinical focus of brief psychodynamic support may be less on stabilizing a fragile self and more on facilitating mourning and integration. Specifically, psychotherapy can provide a structured space to process losses related to pain, sexuality and fertility concerns, integrate chronic illness into the patient’s life narrative, and support renegotiation of identity, intimate relationships and relational roles. This framing is consistent with the SWAP pattern observed in our sample and with the selective reduction in ANPS SADNESS and should be tested in future studies using direct clinical outcomes (e.g., pain and quality of life) and process measures.

### 4.4 Limitations and future directions

Several limitations should be noted. First, the sample was small (*N* = 17), limiting statistical power and the precision of effect sizes and correlations; ANPS results, especially for scales other than SADNESS, should be viewed as preliminary and hypothesis-generating. Second, SWAP-200 was assessed only at baseline, preventing analyses of personality change. Third, the study lacked a control/comparison condition; changes in SADNESS cannot be unequivocally attributed to psychotherapy, as regression to the mean, natural adaptation, or concurrent medical treatments may have contributed. Accordingly, given the uncontrolled, exploratory design, findings should be interpreted as hypothesis-generating rather than confirmatory. Fourth, psychiatric comorbidity and trauma history were derived from routine clinical records rather than standardized assessments, so we did not report detailed diagnostic subtypes or trauma exposure; future studies should include systematic, validated measures to increase clinical granularity. The same situation emerges considering time from symptom onset to endometriosis diagnosis (diagnostic delay), as it was not systematically captured in the available database; given the small sample and marked heterogeneity (ranging from months to years), we were unable to classify participants reliably on this dimension. Fifth, comparisons with the FND cohort relied on separate, non-randomized samples with different sizes, sex composition, and clinical contexts, using summary statistics rather than pooled raw data; therefore, observed similarities (e.g., selective SADNESS reduction; convergence between internalizing SWAP profiles and elevated FEAR/SADNESS) and differences (e.g., higher SEEKING/FEAR/ANGER and more consistently high-functioning/obsessive SWAP profiles in endometriosis vs. more fragile, dependent/avoidant and dysregulated configurations in FND) remain exploratory. Finally, since the analytic sample includes only women who accepted and completed a brief psychotherapy pathway, selection bias is likely; accordingly, findings apply specifically to women with endometriosis who seek and adhere to psychological support in a tertiary-care setting, rather than to the broader endometriosis population. Henceforth, this selection process may also have contributed to the predominance of the aforementioned SWAP profile in completers.

Future research should replicate these findings in larger, prospectively designed samples, ideally including multiple diagnostic groups (e.g., endometriosis, FND, other chronic pain syndromes) within the same analytic framework, with longer follow-up to test durability and whether changes in primary emotional systems mediate improvements in pain, HRQoL, and interpersonal functioning. A feasible and ethically acceptable next step would be a two-arm waitlist design, randomly allocating participants to immediate treatment versus delayed-start, allowing treated–untreated comparisons over the same time window while minimizing concerns about withholding care. Finally, integrating patient-reported outcomes with clinician-rated personality measures and process variables (e.g., alliance, emotional processing) may clarify mechanisms, and controlled studies should include direct clinical outcomes (e.g., pain intensity and health-related quality of life) alongside affective measures to test whether observed changes also consolidate the qualitative impressions from clinical interviews.

## Data Availability

The raw data supporting the conclusions of this article will be made available by the authors, without undue reservation.
